# Supplementary material for: Structure, dynamics, and molecular inhibition of the Staphylococcus aureus m1A22-tRNA methyltransferase TrmK
Source: J Biol Chem. 2022 May 17;298(6):102040. doi: 10.1016/j.jbc.2022.102040 (PMC9190014; doi:10.1016/j.jbc.2022.102040)
Supplement: Supporting information [file mmc1.pdf]

Supporting information

Structure, dynamics, and molecular inhibition of the *Staphylococcus aureus* m^{1}A22-tRNA methyltransferase TrmK

Pamela Sweeney[1], Ashleigh Galliford[1], Abhishek Kumar[2], Dinesh Raju[2], Naveen B. Krishna[2], Emmajay Sutherland[1], Caitlin J. Leo[1], Gemma Fisher[1], Roopa Lalitha[2], Likith Muthuraj[2], Gladstone Sigamani[2], Verena Oehler[1], Silvia Synowsky[1], Sally L. Shirran[1], Tracey M. Gloster[1], Clarissa M. Czekster[1], Pravin Kumar[2,*], and Rafael G. da Silva[1,*]

[1]School of Biology, Biomedical Sciences Research Complex, University of St Andrews, St Andrews KY16 9ST, UK.

[2]Kcat Enzymatic Private Limited, Bangalore, India.

List Of Supporting Figures and Tables:

Figure S1. SDS-PAGE analysis of *Sa*TrmK purification
Figure S2. ESI-TOF-MS analysis of purified *Sa*TrmK
Figure S3. DSF-based thermal denaturation assay of *Sa*TrmK
Figure S4. Determination of the oligomeric state of *Sa*TrmK in solution by analytical gel filtration
Table S1. X-ray data processing and refinement statistics
Figure S5. Overlay of *Sa*TrmK, *Sa*TrmK:SAM, and *Sa*TrmK:SAH over all Cα atoms
Figure S6. Overlay of Cα atoms of *Sa*TrmK, *B. subtilis* TrmK (PDB entry 6Q56), and *S. pneumoniae* TrmK (PDB entry 3KR9)
Figure S6. Overlay of Cα atoms of *Sa*TrmK, *B. subtilis* TrmK (PDB entry 6Q56), and *S. pneumoniae* TrmK (PDB entry 3KR9)
Figure S7. Possible interaction between *Sa*TrmK and citrate
Figure S8. Position of Asp26 side chain in *Sa*TrmK:SAM and *Sa*TrmK apoenzyme
Figure S9. Domain cross-correlation matrices
Figure S10. Molecular electrostatic potential surfaces
Figure S11. LC-MS for SAH detection
Figure S12. Dependence of luminescence counts on SAH concentration
Figure S13. Effect of sinefungin on the quantification of SAH via the MTase-Glo™ Methyltransferase Assay
Figure S14. Effect of plumbagin on the quantification of SAH via the MTase-Glo™ Methyltransferase Assay
Figure S15. Potential covalent adducts of plumbagin with *Sa*TrmK
Table S2. Oligonucleotide primers for production of DNA templates for *in-vitro* transcription

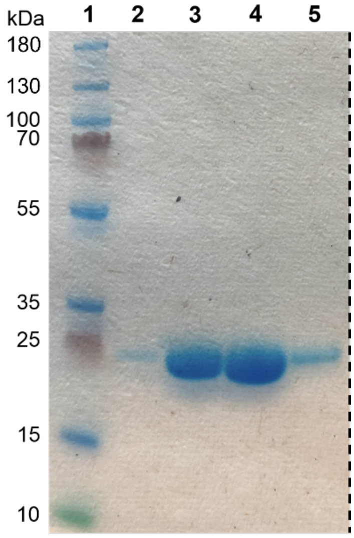

Figure S1. SDS-PAGE analysis of *Sa*TrmK purification. Lane 1, Thermo Scientific Page Ruler Plus Prestained Protein Ladder. Lane 2 – 5, flowthrough from the HisTrap FF 5 mL column after TEVP cleavage. The dashed line indicates where the image was cropped after lane 5 since the remaining lanes had not been loaded with sample.

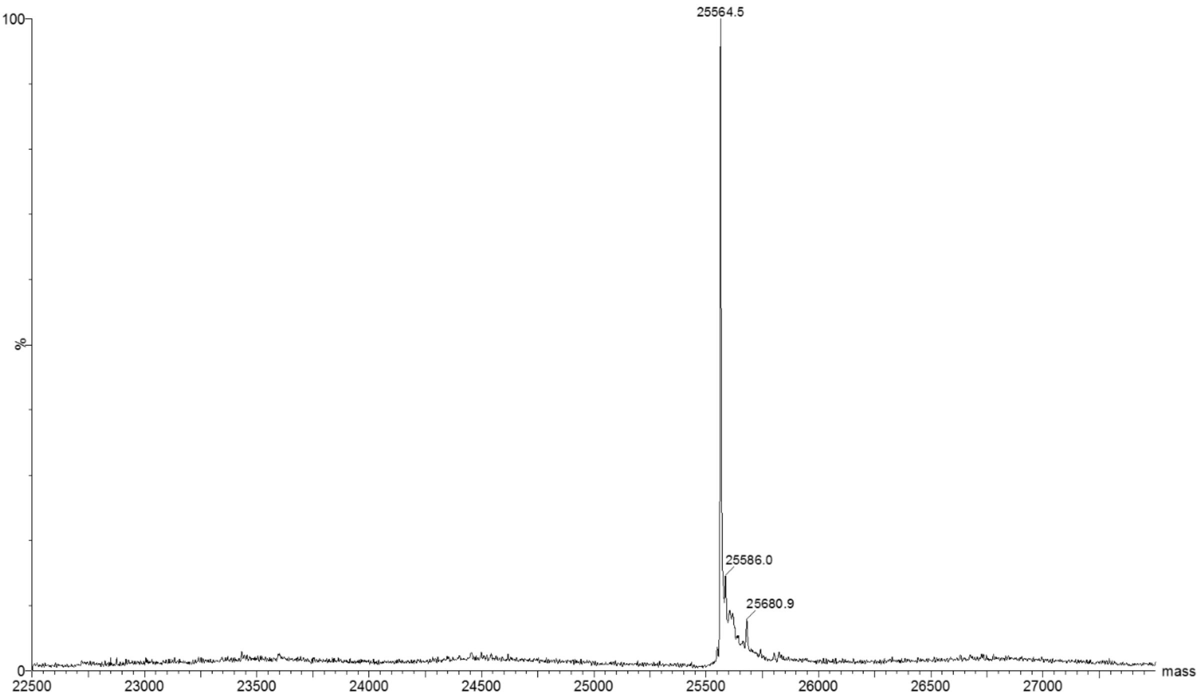

Figure S2. ESI-TOF-MS analysis of purified *Sa*TrmK. The experimental mass matches the predicted molecular mass of 25,565.3.

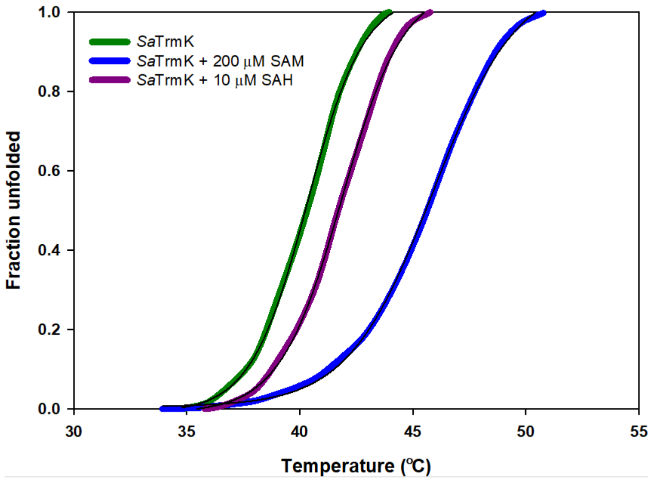

Figure S3. DSF-based thermal denaturation assay of *Sa*TrmK. Lines of best fit to equation 2 are in black.

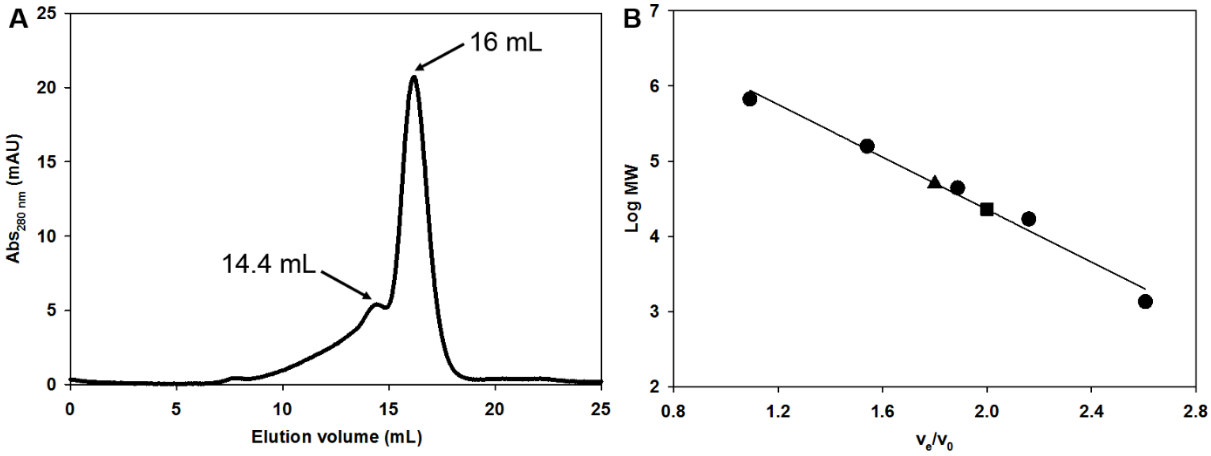

Figure S4. Determination of the oligomeric state of *Sa*TrmK in solution by analytical gel filtration. **(A)** Elution profile of *Sa*TrmK. **(B)** Relationship between molecular weight and elution volume (v_e) to void volume (v_0) ratio using molecular weight standards (circles). The line is a linear regression of the data. The v_e/v_0 ratio involving the major and minor elution volumes of *Sa*TrmK are plotted as square and triangle, respectively.

Table S1. X-ray data processing and refinement statistics.

	*Sa*TrmK apo	*Sa*TrmK:SAM	*Sa*TrmK:SAH
**PDB**	**7O4M**	**7O4N**	**7O4O**
**Wavelength (Å)**	0.9795	0.9795	0.9795
**Resolution range (Å)**	43.8 - 1.30 (1.32 - 1.30)	43.4 - 1.40 (1.45 - 1.40)	19.4 - 1.52 (1.57 - 1.52)
**Space group**	*P* 2_1 2_1 2_1	*P* 2_1 2_1 2_1	*P* 2_1 2_1 2_1
**Unit cell dimensions**			
**a, b, c (Å)**	59.23, 60.71, 63.13	59.64, 61.31, 63.28	59.75, 61.44, 63.19
**α, β, γ (°)**	90, 90, 90	90, 90, 90	90, 90, 90
**Total reflections**	273800 (13644)	91928 (8937)	71273 (7033)
**Unique reflections**	56680 (2836)	46295 (4497)	36225 (3577)
**Multiplicity**	4.8 (4.8)	2.0 (2.0)	2.0 (2.0)
**Completeness (%)**	99.9 (99.9)	99.5 (98.8)	99.3 (99.3)
**Mean I/sigma(I)**	17.5 (1.9)	13.38 (1.52)	15.60 (1.54)
**R-merge**	0.04 (0.251)	0.039 (0.68)	0.034 (0.48)
**CC1/2**	0.99 (0.94)	0.99 (0.44)	0.99 (0.39)
**Reflections used in refinement**	53540 (3928)	46146 (4497)	36225 (3576)
**R-work**	0.115	0.148	0.169
**R-free**	0.148	0.199	0.231
**RMSD (bonds)**	0.013	0.011	0.011
**RMSD (angles)**	1.77	1.61	1.70
**Number of non-hydrogen atoms**	2355	2197	2150
**Protein**	1954	1880	1839
**Ligands**	19	33	38
**Solvent**	382	284	273
**Average B-factor ($Å^2$)**	16.9	19.9	20.6
**Protein**	14.3	17.6	18.5
**Ligands**	18.5	22.8	19.9
**Solvent**	29.9	35.2	34.8
**Ramachandran favoured (%)**	99	98	98
**Ramachandran allowed (%)**	1	2	2
**Ramachandran outliers (%)**	0	0	0

Values within brackets are for the highest resolution shell.

RMSD, root mean square deviation

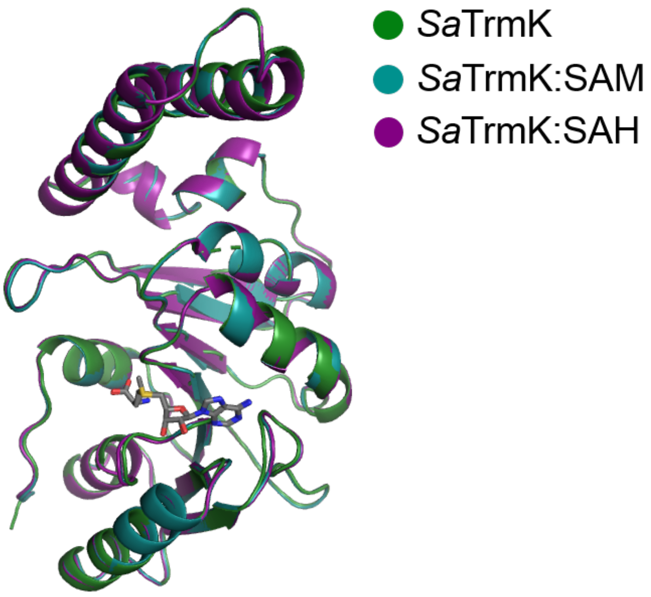

Figure S5. Overlay of *Sa*TrmK, *Sa*TrmK:SAM, and *Sa*TrmK:SAH over all Cα atoms.

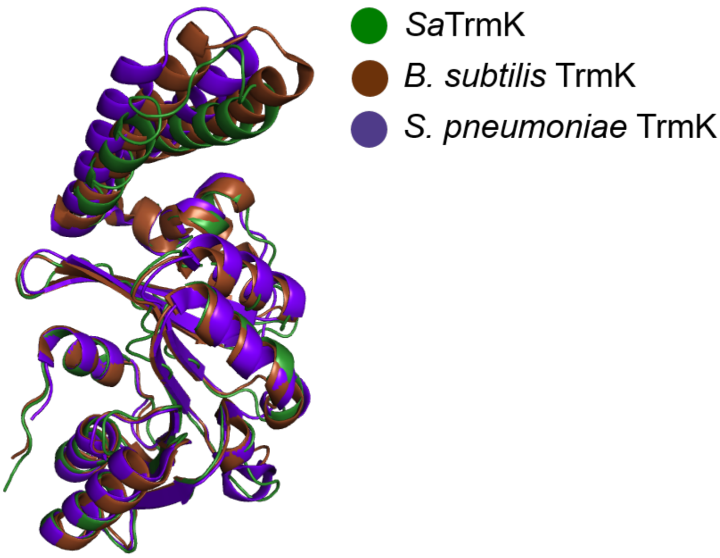

Figure S6. Overlay of Cα atoms of *Sa*TrmK, *B. subtilis* TrmK (PDB entry 6Q56), and *S. pneumoniae* TrmK (PDB entry 3KR9).

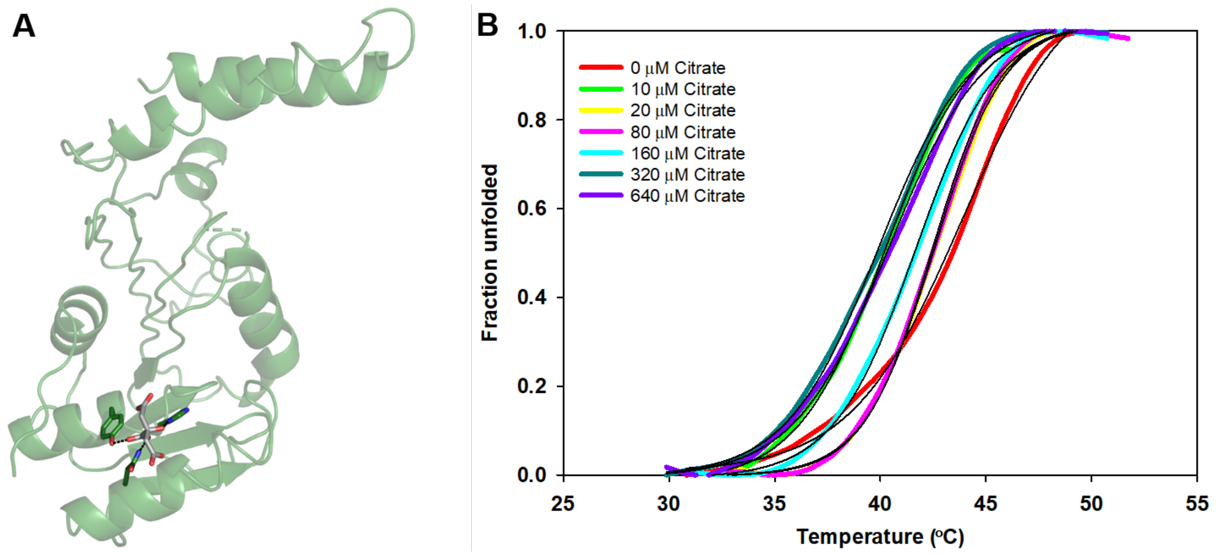

Figure S7. Possible interaction between *Sa*TrmK and citrate. (A) Polar contacts (dashed lines) among citrate and the side chains of His27, Tyr29, and Asn59. Side chains (green) and citrate (grey) are depicted as stick models. **(B)** DSF-based thermal denaturation assay of *Sa*TrmK in the presence and absence of citrate. Lines of best fit to equation 2 are in black, yielding T_m (°C) of 43.0 ± 0.2, 40.2 ± 0.1, 42.5 ± 0.1, 42.04 ± 0.06, 41.67 ± 0.08, 39.9 ± 0.09, 40.4 ± 0.1, respectively, from 0 – 640 µM citrate.

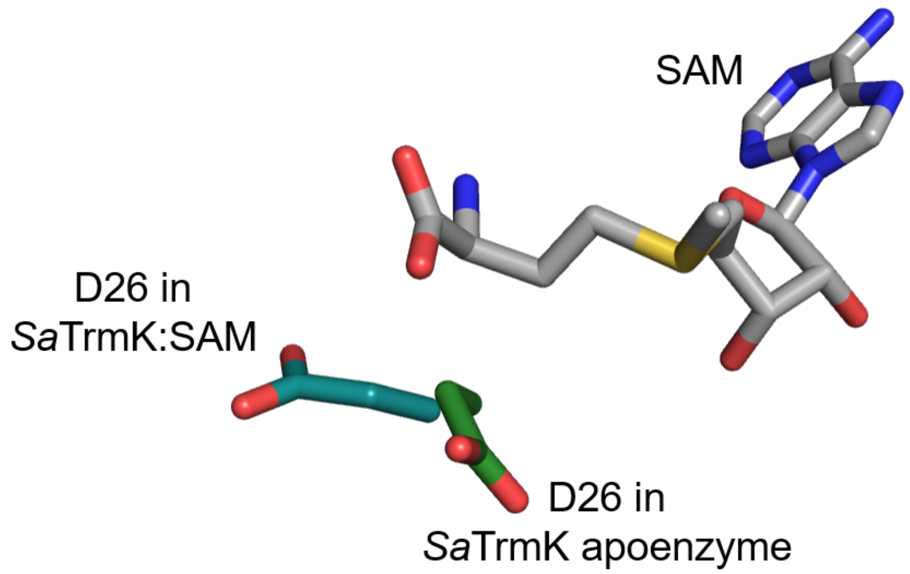

Figure S8. Position of Asp26 side chain in *Sa*TrmK:SAM and *Sa*TrmK apoenzyme. Asp26 side chain and SAM are shown in stick models.

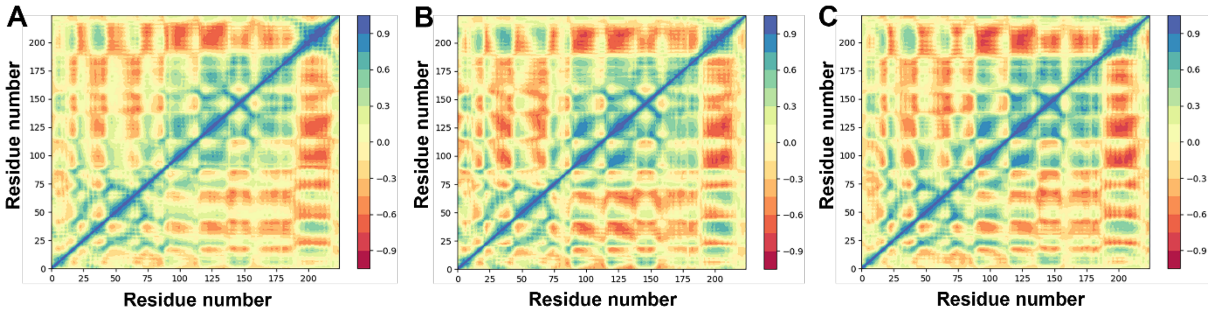

Figure S9. Domain cross-correlation matrices. (A) *Sa*TrmK apoenzyme. **(B)** *Sa*TrmK:SAM. **(C)** *Sa*TrmK:SAH.

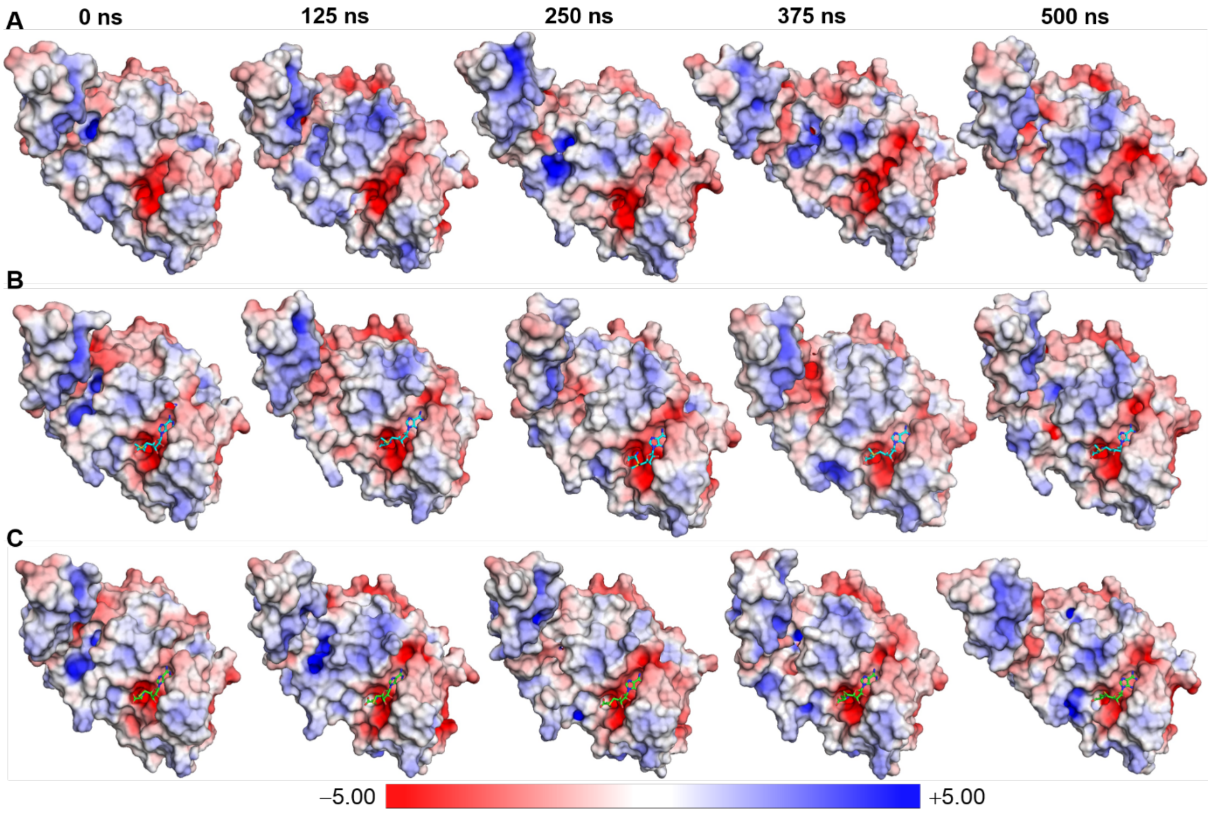

Figure S10. Molecular electrostatic potential surfaces. (A) Time-dependent molecular electrostatic potential surface of *Sa*TrmK. **(B)** Time-dependent molecular electrostatic potential surface of *Sa*TrmK:SAM, where SAM is shown as stick model. **(C)** Time-dependent molecular electrostatic potential surface of *Sa*TrmK:SAH, where SAH is shown as stick model. For all surfaces, snapshots of the electrostatic potential were taken at the times indicated at the top along the 500 ns of MD simulations. The units of the colour saturation are kT/e.

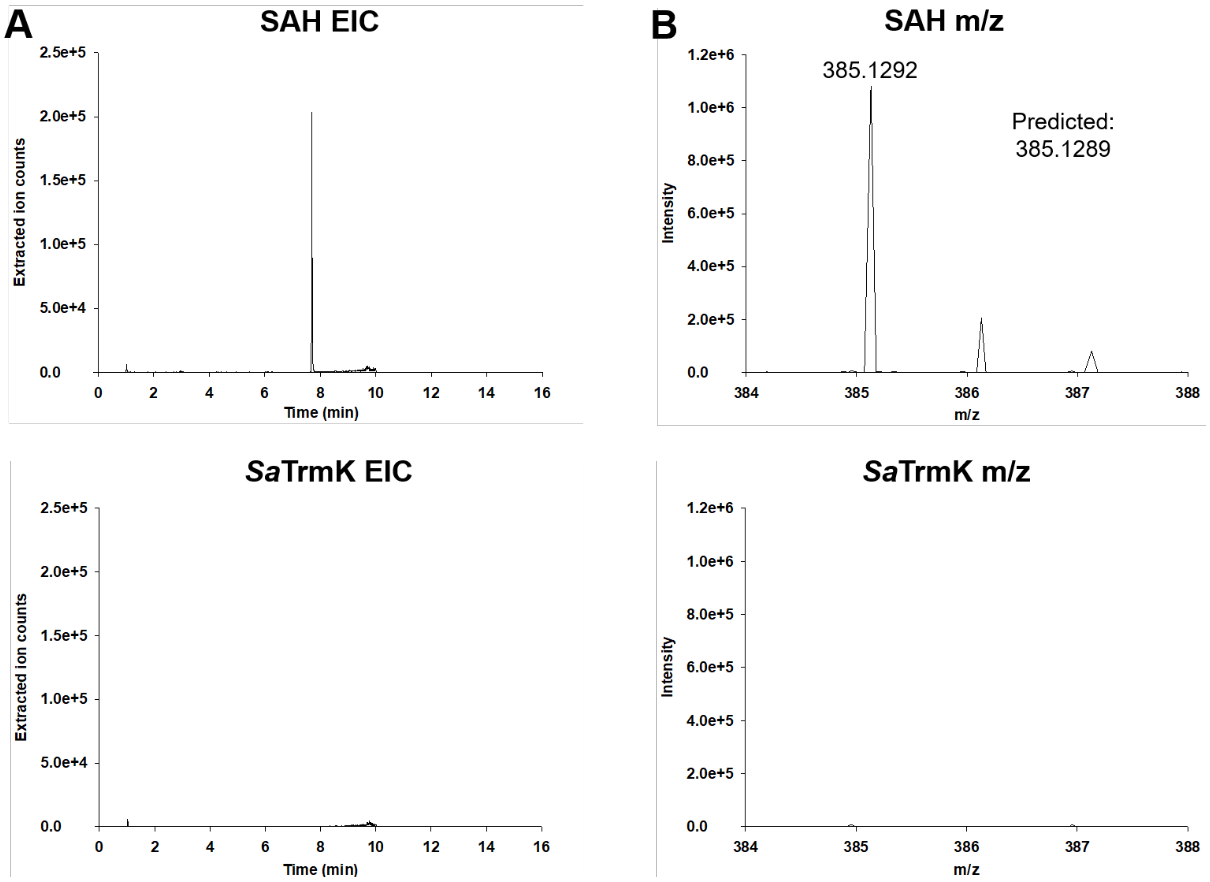

Figure S11. LC-MS for SAH detection. (A) Extracted ion counts of TCA-treated SAH and TCA-treated SaTrmK. **(B)** Detected mass from the respective extracted ion counts.

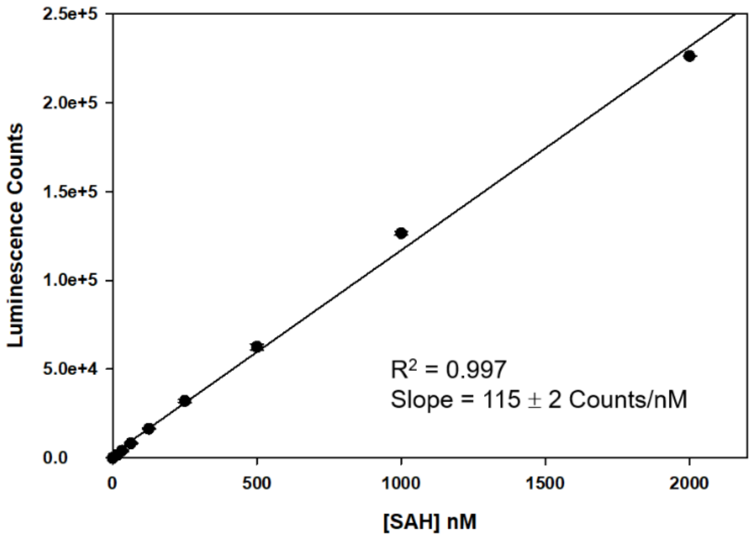

Figure S12. Dependence of luminescence counts on SAH concentration. Data are mean ± SD of duplicate measurements. The line is a linear regression of the data.

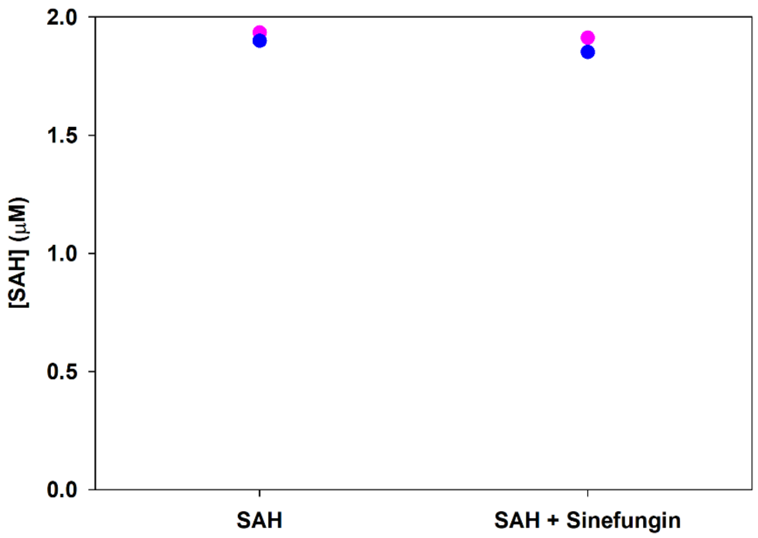

Figure S13. Effect of sinefungin on the quantification of SAH via the MTase-Glo™ Methyltransferase Assay. Each data point of the duplicate measurements is shown (pink and blue).

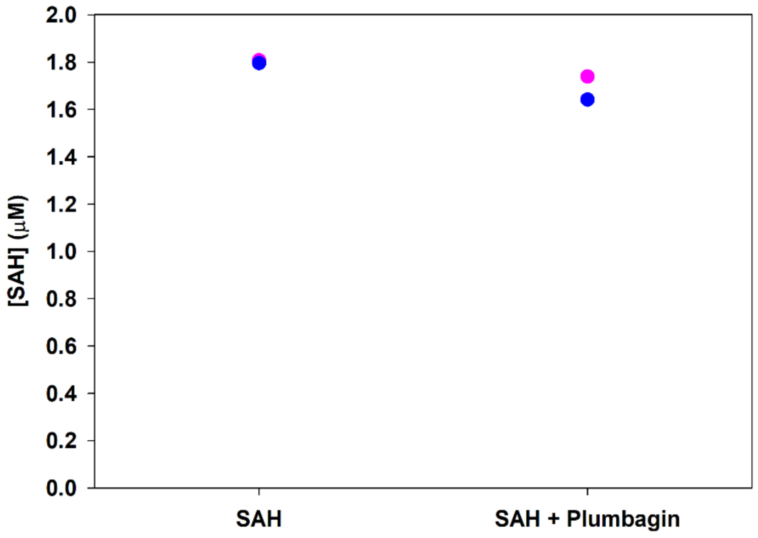

Figure S14. Effect of plumbagin on the quantification of SAH via the MTase-Glo™ Methyltransferase Assay. Each data point of the duplicate measurements is shown (pink and blue).

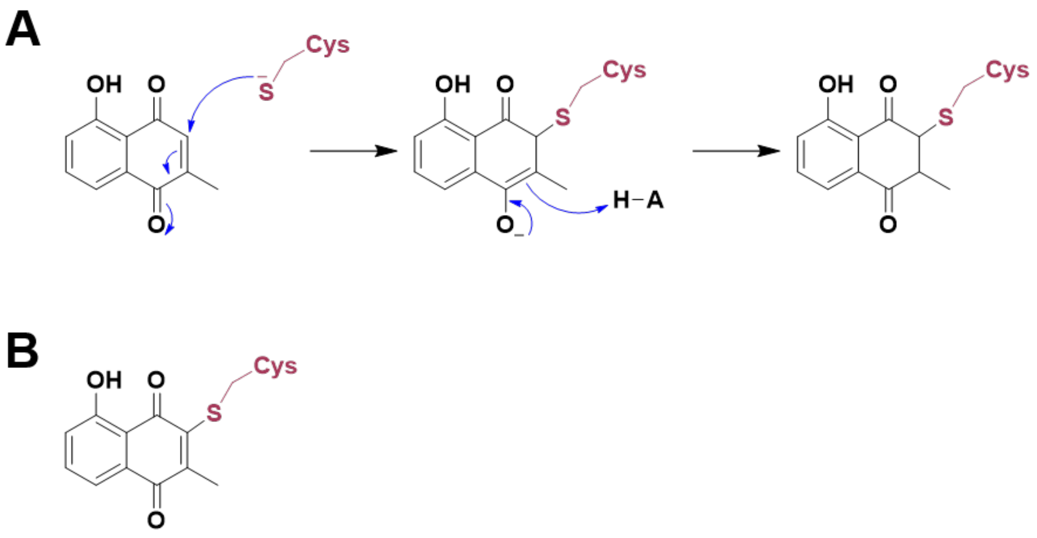

Figure S15. Potential covalent adducts of plumbagin with *Sa*TrmK. (A) The mechanism of Michael addition. The final Michael adduct would produce a mass difference of 188 in comparison with the unlabelled enzyme. **(B)** Putative oxidation product following Michael addition, which would result in a mass difference of 186 in comparison with the unlabelled enzyme.

Table S2. Oligonucleotide primers for production of DNA templates for *in-vitro* transcription.

Amplicon	Primers (5′ to 3′)
**tRNA^{Leu}**	Forward Primer 1: CTCGAGTAATACGACTCACTATAGGCGGTCGTGGCGGAA<br>Reverse Primer 2: TGGTGGATGCGGCCGAG
**A22C-tRNA^{Leu}**	Forward Primer 1: CTCGAGTAATACGACTCACTATAGGCGGT
	Reverse Primer 2: AGGCCCTCAACCTAGCGCAGCTGCCATTCCGCCACGACCGCCTATAGTGAGTCGTATTA
	Forward Primer 3: GCTAGGTTGAGGGCCTAGTGGGAGAAGTCCCGTGGAGGTTCAAGTCCTCTCGGCCGCATC
	Reverse Primer 4: TGGTGGATGCGGCCGAGAG
**A22U-tRNA^{Leu}**	Forward Primer 1: CTCGAGTAATACGACTCACTATAGGCGGTCGTGGCGGAATGGCAGTTGCGCT
	Reverse Primer 2: CCCACTAGGCCCTCAACCTAGCGCAACTGCCATTC
	Forward Primer 3: TTGAGGGCCTAGTGGGAGAAGTCCCGTGGAGGTTCAAGTCCTCTCGGCCGCATC
	Reverse Primer 4: TGGTGGATGCGGCCGAGAG
**A22G-tRNA^{Leu}**	Forward Primer 1: CTCGAGTAATACGACTCACTATAGGCGGTCGTGGCGGAATGGCAGGTGCGCT
	Reverse Primer 2: CCCACTAGGCCCTCAACCTAGCGCACCTGCCATT
	Forward Primer 3: TTGAGGGCCTAGTGGGAGAAGTCCCGTGGAGGTTCAAGTCCTCTCGGCCGCATC
	Reverse Primer 4: TGGTGGATGCGGCCGAGAG
**RNA^{18mer}**	CTCGAGTAATACGACTCACTATAGGCGGTCGGCAACGACCGC
**A10G-RNA^{18mer}**	CTCGAGTAATACGACTCACTATAGGCGGTCGGCGACGACCGC
**A11G-RNA^{18mer}**	CTCGAGTAATACGACTCACTATAGGCGGTCGGCATCGACCGC

**U5C/A14G-RNA^{18mer}**	CTCGAGTAATACGACTCACTATAGGCGGCCGGCAACGGCCGC
**A11-RNA^{18mer}**	CTCGAGTAATACGACTCACTAGGCGGGCGGCGACGCCCGC
**0A-RNA^{18mer}**	CTCGAGTAATACGACTCACTAGGCGGGCGGCGGCGCCCGC
